# Study of the collagen structure in the superficial zone and physiological state of articular cartilage using a 3D confocal imaging technique

**DOI:** 10.1186/1749-799X-3-29

**Published:** 2008-07-17

**Authors:** Jian P Wu, Thomas B Kirk, Ming H Zheng

**Affiliations:** 1 3D imaging laboratory, the School of Mechanical Engineering, The University of Western Australia, Perth, WA, Australia; 2 Orthopaedic Surgery, School of Surgery and Pathology, The University of Western Australia, Perth, WA, Australia

## Abstract

**Introduction:**

The collagen structure in the superficial zone of articular cartilage is critical to the tissue's durability. Early osteoarthritis is often characterized with fissures on the articular surface. This is closely related to the disruption of the collagen network. However, the traditional histology can not offer visualization of the collagen structure in articular cartilage because it uses conventional optical microscopy that does not have insufficient imaging resolution to resolve collagen from proteoglycans in hyaline articular cartilage. This study examines the 3D collagen network of articular cartilage scored from 0 to 2 in the scoring system of International Cartilage Repair Society, and aims to develop a 3D histology for assessing early osteoarthritis.

**Methods:**

Articular cartilage was visually classified into five physiological groups: normal cartilage, aged cartilage, cartilage with artificial and natural surface disruption, and fibrillated. The 3D collagen matrix of the cartilage was acquired using a 3D imaging technique developed previously. Traditional histology was followed to grade the physiological status of the cartilage in the scoring system of International Cartilage Repair Society.

**Results:**

Normal articular cartilage contains interwoven collagen bundles near the articular surface, approximately within the lamina splendens. However, its collagen fibres in the superficial zone orient predominantly in a direction spatially oblique to the articular surface. With age and disruption of the articular surface, the interwoven collagen bundles are gradually disappeared, and obliquely oriented collagen fibres change to align predominantly in a direction spatially perpendicular to the articular surface. Disruption of the articular surface is well related to the disappearance of the interwoven collagen bundles.

**Conclusion:**

A 3D histology has been developed to supplement the traditional histology and study the subtle changes in the collagen network in the superficial zone during early physiological alteration of articular cartilage. The fibre confocal imaging technology used in this study has allowed developing confocal arthroscopy for *in vivo *studying the chondrocytes in different depth of articular cartilage. Therefore, the current study has potential to develop an *in vivo *3D histology for diagnosis of early osteoarthritis.

## Background

The structure and composition of articular surface are controversy topics in the literature. Lamina splendens was described as a bright layer covering on the top of articular cartilage (AC) by MacConeil using phase contrasted microscopy [1]. It was then argued as an artifact generated by phase contrasted microscopy [2]. Transmission electron microscopy [3,4] and scanning electron microscopy (SEM) [5] confirmed the existent of the lamina splendens. Thereafter, some scholars suggested that lamina splendens contained collagen fibres [6,7] while others argued the lamina splendens is amorphous [8]. Confocal microscopy reported that a semitransparent membrane on the top of articular cartilage could be physically peeled off from the rest of AC and contained unique collagen network [9].

Collagen possesses great tensile strength. It forms a 3D network in AC which constrains the swelling pressure of hydrated proteoglycans and contributes in the configuration of the unique mechanical properties of AC. The top 10% of cartilage thickness is often referred as the superficial zone [10,11] where the orientation of the collagen fibres is particularly important to tensile strength of the articular surface and durability of AC [12,13]. The collagen fibres in the superficial zone have also been traditionally suggested to align predominantly in a direction parallel to articular surface [14]. The elastic modulus of the superficial zone has been reported to be 7 GPa and 2.21 GPa in the directions parallel and perpendicular to the cleavage line pattern respectively [15].

Early OA is characterized with lesions of articular surface [16,17], closely associated with disruption of the collagen fibres and network in the superficial zone [18-21]. Loss of the most superficial layer of AC has been reported to lead to rapid wear of the AC, and consequently reduction of the loading capacity of AC as a result of progressive release of proteoglycans from the cartilage [22]. Therefore, study of the 3D collagen network in AC offers to understand the early event involved in degeneration of AC and OA [23,24]. It will greatly assist developing a technique to detect early physiological changes in AC.

Traditional histology is used as a method to study the physiological condition of AC and obtain OA grade [25]. However, this technique often uses optical microscopy, which does not have an ability to image the collagen structure in AC [26]. Consequently, it can not be used to detect the disruption of the collagen network in the superficial zone that fundamentally leads to the lesion of articular surface and early OA. CT, ultrasound and MRI also provide a way to study the degeneration of AC and OA but these imaging techniques do not have sufficient imaging resolution to study the microstructure of AC and detecting OA at an early development period. Electron microscopy (EM) is the only imaging technique that has sufficient image resolution for studying the more detailed collagen structure in AC. However, this technique requires special imaging environments and tissue preparation, which not only cause artifacts but also restrict its clinical applications. Most of all, all these imaging techniques are fundamentally limited in 2D observations. For stereological study, the AC must be physically sectioned and dehydrated to obtain a series of 2D images before a complex computer program is used to reconstruct them as a 3D image.

Confocal microscopy has a higher image resolution than conventional light microscopy. It also allows study of the internal microstructure of bulk AC without dehydrating and physically sectioning the tissue. Therefore, artifacts associated with sample dehydration and sectioning are largely eliminated. Fibre optic laser confocal microscopy uses an optic fibre to perform the function of a pinhole in a conventional confocal microscope to obtain images of bulk biological tissues [27]. The optic fibre imaging technology which it uses has permitted the development of confocal arthroscopy to work as a way of optical histology for study of the cellular morphology in different depth of AC *in vivo *[28-30] but the collagen network in AC has not been resolved. Using a specific dye for collagen (type I, II and III) and the fibre optic laser scanning confocal microscopy, a 3D imaging technique has been developed previously and used successfully for study of the 3D collagen structure up to about 80 μm deep from the articular surface [9]. Using this 3D imaging technique, the present study examines the 3D collagen network in the AC with different physiological status, scored in the International Cartilage Repair Society (ICRS) Grading System from ICRS 0 to ICRS 3. A 3D histology has been developed to study the 3D collagen structure in the superficial zone by means of predicting potential surface lesions and diagnosing early OA.

## Method

### Specimens

**Figure 1:**
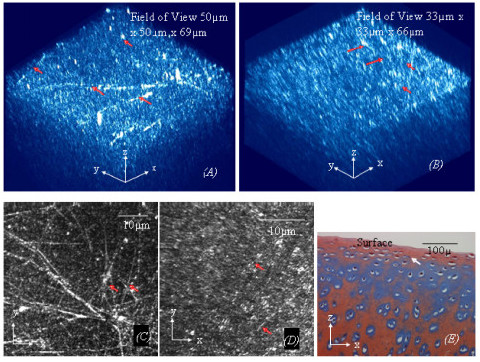
***(A)*****. A 3D image of normal cartilage (ICRS grade 0, shown in Fig. 1(*****E*****) from a cow femoral head at a lower magnification view using digital zooming shows more clearly that the structure of the interwoven collagen bundles (ICB) near the articular surface (the arrows in Fig 1(*****A*****)).***(B)*. A 3D image of normal cartilage from a cow femoral condyle at a higher magnification view using digital zooming shows more clearly the orientation of the collagen fibres in the superficial zone, which is align predominantly in a spatial direction oblique the AC surface. *(C): *MBI reconstructed from the top eight of 2D images in Fig 1*(A*) shows clearly the interwoven collagen bundles near the articular surface. (*D): *The MBI reconstructed from the image stack used to reconstruct the 3D image in Fig 1(*B*) is analogous to an *en face *2D observation, by which the collagen fibres in the superficial zone appear to align predominantly in a direction parallel to the AC surface in 2D images. *(E*): The corresponding traditional histology of a cow femoral condyle used for ICRS grading shows proteoglycans (blue) are highly deposited in normal cartilage.

Cylindrical cartilage specimens, about 3 mm diameter attached to the subchondral bone, were obtained from five categories of physiological status according to their macroscopic appearance under the supervision of orthopaedic surgeons. Forty-three normal cartilage specimens (N) were cut from central loading regions of ten femoral condyle and five femoral heads of approximately two-year-old cows within 24 hours of slaughter. Using the technique developed previously [9], another fifteen normal cartilage specimens were peeled off the most superficial semitransparent membrane corresponding to the lamina splendens to create articular surface disruption (as shown in Fig. 1). Twenty-two aged cartilage (AG) specimens, which demonstrated little surface disruption, were obtained from five femoral heads of human cadavers aged from 40 to 60 years old. Twenty-eight cartilage specimens (SD) were obtained from regions that showed slightly surface disruption of fifteen human arthritic femoral heads from joint replacement surgery. Six fibrillated cartilage specimens (F) were harvested from regions that displayed distinctive surface lesions of the human arthritic femoral heads from joint replacement surgery. The normal cartilage samples were selected from both lateral and medial regions. The aged cartilage samples were carefully selected from the regions with little surface lesions, therefore, majority of the samples in this physiological group were from the central loading regions but some of them were from unloaded regions. Cartilage samples with minor natural surface lesion were randomly selected from the regions of the femoral heads without a serious OA invasion. However, during imaging the details of the sample location were not lodged.

All specimens were fixed in 10% buffered formalin solution (BFS) for 24 hours, and immersed into 0.2% Phosphomolybdic acid solution for another 24 hours at 4°C before stained with 1 g/L Picrosirius red for 72 hours. After being washed in 9 g/L saline solution, the specimens were put into specially designed specimen dishes to maintain their hydrated state and acquire collagen images using a fibre optic laser scanning confocal microscope (FOCM, Optiscan Pty Ltd, Melbourne, Australia).

### Imaging techniques

Prior to image acquisition, the FOCM was calibrated by using focal check fluorescent microspheres (Molecular Probes, The Netherlands). An optimal image stack of the collagen fibres was acquired up to a depth of 80 μm from the articular surface by use of an Olympus PlanApo 60×/1.4 oil immersion lens through a reflectance channel illuminated by 488 nm (50%) and 514 nm (50%) lasers. This provided a 0.23 μm lateral resolution and 0.73 μm depth resolution. The optical sectioning size was set at 0.541 or 0.689 μm. The magnification can be changed from low to high using the digital zooming function within computer software F900e, proprietary to the confocal microscope. However, the magnifications at a field view of 50 μm × 50 μm (low magnification) and 33 μm × 33 μm (high magnification) are used in this study to produce optimal observations of the collagen orientation. Using computer software VoxBlast (VayTek, Inc, USA), the image stack of the collagen fibres was reconstructed as a 3D image for visual inspections. Using F900e, the image stack was also processed to provide a maximum brightness image (MBI), which contains the maximum pixel value for each xy location from all the 2D image slices and is analogous to an *en face *image (parallel to the articular surface) in 2D microscopy.

### Traditional histology and International Cartilage Research Society grading

Alcian Blue stains proteoglycans (PGs) of AC as blue [31]. Following the confocal microscopic imaging, a traditional histology image using Alcian Blue staining was obtained to grade the physiological status of AC in terms of International Cartilage Research Society (ICRS) scores and understand the approximate concentration of the PGs in AC. An optical microscopy (Zeiss Axioplan 2) was used. Therefore, the relationship between the 3D collagen structure and physiological condition of AC can be studied.

After decalcified in 5% formic acid for about 7 days to soften the subchondral bone and washed thoroughly in tap water, the AC specimens were sliced approximately as 5 μm thick sections by microtome. The slices were rinsed in 3% acetic acid and stained by 1% Alcian Blue 8 GX (C.I74240, Scot Scientific, Australia) for 10 minutes. They were rinsed in tap water followed by a nuclei counterstained for 1 minutes using 0.5% Safranine O (C.I.50240, Hopkin & Willianms, England). After quickly rinsed in tap water, the slices were washed in 70%, 95%, and three changes of 100% ethanol. After this, the slices were washed by three changes of 100% xylene before embedded on glass slides.

## Results

The collagen fibres in the superficial zone of AC form a 3D microstructure that is much more complex than has been described by previous 2D microscopic studies. The 3D collagen structure alters with both the age and physiological status of AC, as shown in Figs 1, 3, 4, 5. Normal cartilage, ICRS grade 0, shown in Fig 1(E), is distinguished by unique interwoven collagen bundles running near the articular surface, as shown in Figs 1(A)–(D). However, the collagen fibres in the superficial zone are predominantly oriented in a direction spatially oblique to the articular surface in a detailed 3D observation, as shown in Fig 1(B). Despite displaying an oblique orientation in a spatial presentation, the collagen fibres in the superficial zone appear to be oriented predominantly in a direction parallel to the AC surface in the corresponding MBI that is analogous to an *en face *2D observation, as shown in Fig 1(D). Clearly, the characteristics of the interwoven collagen bundles are shown more prominent at low magnification (Fig 1(A)) and a MBI that is only reconstructed by the 2D image slices near the articular surface (Fig 1(C)); whereas, the orientation of the oblique collagen fibres is seen more clearly at higher magnification (Fig 1(B)). Traditional histological studies using Alcian Blue stain show that proteoglycans are highly concentrated within the normal AC, as shown in Fig 1(E).

**Figure 2:**
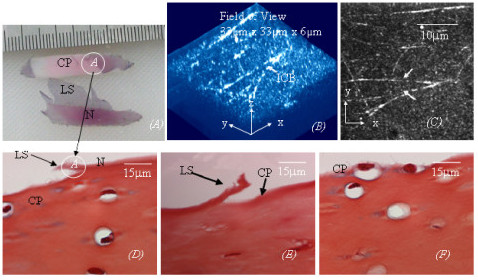
***(A)*****. A semitransparent membrane corresponding to the lamina splendens (LS) was physically peeled off from normal articular cartilage (N) of a cow femoral head (unloading region).***(B)*. A 3D image of the lamina splendens shows the collagen network within it is compromised of unique interwoven collagen bundles (ICB). *(C)*. The corresponding MBI of the collagen network in LS in Fig *(B*). *(D)*. Traditional histology shows the site where the lamina splendens was separated from the normal (cow) cartilage. *(E)*. Traditional histology of early arthritic cartilage from a human femoral head shows disrupting the articular surface in early OA is a process similar to physically peeling off the lamina splendens. *(F)*. Traditional histology of normal cartilage physically peeled the lamina splendens (indicated as CP (cartilage peeled lamina splendens) in Fig 2*(A*)) shows loss of the most superficial layer of articular cartilage can expose some chondrocytes near the surface to the joint cavity.

**Figure 3:**
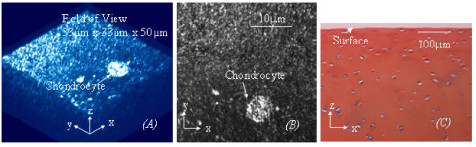
***(A)*****. In approximately half of aged cartilage specimens (from cadaver femoral heads) with little surface lesion (ICRS Grade 0, shown in Fig 3*****(C*****)), the collagen fibres in the superficial zone are oriented predominantly in a direction spatially oblique to the AC surface.** However, the fibres are rarely integrated the interwoven collagen bundles on the surface. *(B)*. The corresponding MBI of the collagen network is analogous to an *en face *2D image. *(C*). Traditional histology shows the cartilage is almost at ICRS grade 0 but it contains less proteoglycans than the normal cartilage.

**Figure 4:**
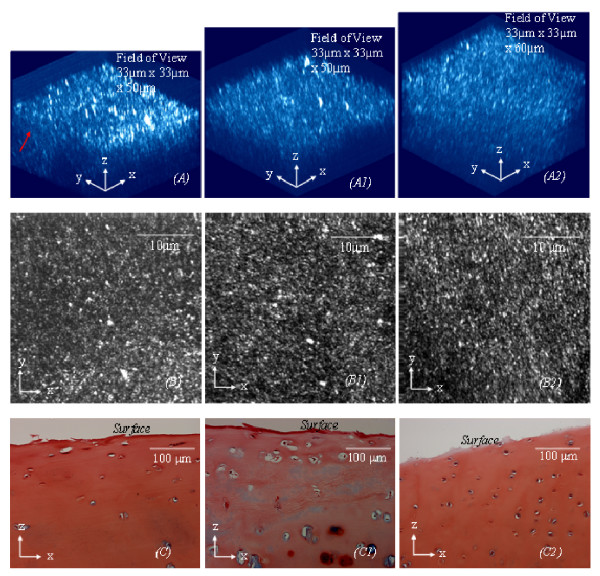
**Approximately another fifty percent of the aged specimens (Fig 4*****(C*****)) from human femoral heads display a similar physiological condition (approximate ICRS Grade 1) to a small proportion of arthritic cartilage specimens (Figs 4(*****C1***)**) from human femoral heads and the cartilage (from cow femoral heads) physically peeled off the lamina splendens (Fig 4*****(C2*****)).** These cartilage specimens, as shown in Figs *(A*), *(A1*) and (*A*2), also have a 3D collagen structure similar to each other and contain the collagen fibres that oriented predominantly in a spatial direction perpendicular to the AC surface. Figs 4*(B), (B1) and (B2*) are the corresponding MBI images, which are analogous to *enface *2D images. Figs 4*(C*), (*C1*) and (*C2*) are the corresponding histology used for ICRS grading. The field of the 3D collagen network in images

**Figure 5:**
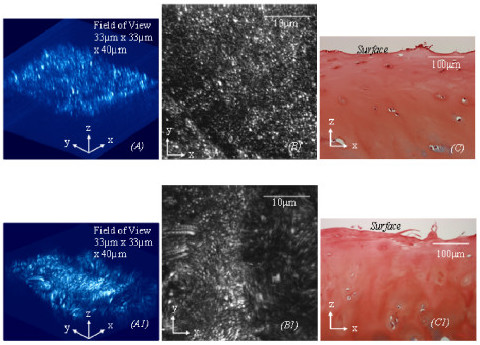
***(A)*****. The 3D collagen network (33 μm × 33 um ×) of the cartilage with a matte surface (ICRS Grade 1–2 in Fig 5*****(C*****)) obtained from human femoral heads is disrupted and compromised of the collagen fibres aligning predominantly in a direction spatially perpendicular to the AC surface. (*****A1)*****.** The 3D collagen network (33 μm × 33 um) of fibrillated cartilage (ICRS grade 3 in Fig 5*(C1*)) has an abnormal microstructure and collagen orientation. Images *(B*)-(*B1*) are the corresponding MBIs of images *(A*) and (*A1*), which are analogous to *en face *2D images. Images *(C)*-*(C1*) are the corresponding histological images used for ICRS grading.

The interwoven collagen bundles observed near the articular surface of normal cartilage have been further confirmed to be within a semitransparent membrane corresponding to the lamina splendens. Therefore, the membrane is provided with considerable tensile strength, which allows it to be differentiated physically from the cartilage, as demonstrated in Figs 2(A) to 2(D). A similar process to the physical delaminating of the most superficial membrane in experiments, as shown in Fig 2(D), has also been observed during early OA degeneration, as shown in Fig 2(E). Further more, physically peeling off the most superficial membrane, comparable to disruption of the articular surface, is able to expose some chondrocytes near the articular surface to the joint cavity, Fig 2(F).

In spite of increase of age, approximately 50% of the cartilage specimens demonstrate little surface disruption (approximate ICRS grade 0), as shown in Fig 3(C). The collagen network also dose not have clearly structural damage, and the fibres in the superficial zone align predominantly in a direction spatially oblique the AC surface, as shown in Fig 3(A). However, the interwoven collagen bundles as seen running near the articular surface of normal AC are rarely found in the aged cartilage, as shown in Fig 3(A). Histological studies using Alcian Blue stain also shows proteoglycans are largely depleted from the aged cartilage, as shown in Fig 3(C). Another 50% of the aged specimens, ICRS grade 1, as shown in Fig 4(C), present surface disruption which is similar to a small proportion of arthritic cartilage (about 4% of the arthritic cartilage specimens) and the cartilage physically peeled off the lamina splendens, as shown respectively in Figs 4(C1) to 4(C2). It is worthy of note that these three types of cartilage also have a collagen network resembling each other, as shown in Figs 4(A) to 4(A2). The interwoven collagen bundles, as seen in normal AC, are totally wiped from the cartilage and the collagen fibres of them are oriented predominantly in a direction spatially perpendicular to the AC surface. Depleting the proteoglycans has also been found in the three types of cartilage, as shown in Figs 4(C) to 4(C2).

A majority of arthritic cartilage specimens (up to 96%) are matte, ICRS grade 1–2, as shown in Fig 5(C). The collagen network of the cartilage in ICRS grade 1–2 presents different levels of structural disruption, and it is constructed by the collagen fibres that were oriented predominantly in a direction spatially perpendicular to the AC surface, as shown in Fig 5(A). In comparison, the fibrillated cartilage in ICRS grade 3, as shown Fig 5(C1), is macroscopically distinguished from the cartilage in ICRS grade 1–2. The collagen fibres of it have different orientation from any other types of the cartilage mentioned previously, as shown in Figs 5(A1) and 5(B1). The fibres in this physiological group did not have preferred orientations in either oblique or perpendicular to articular surface. Excessive damage of the collagen network and torn of the fibres are obviously seen in the specimens. These microscopic features of the collagen network are well correlated to their loss of the lamina splendens status shown by traditional histology, as shown in Fig 5(C1).

## Discussion

Using a 3D imaging technique, this study investigates the 3D collagen structure in the superficial zone in relation to the physiological status of AC. Since the 3D imaging technique does not require physically sectioning and dehydrating the AC, the 3D collagen network revealed in this study closely represents the natural character of the collagen network in AC. Therefore, the changes observed in the 3D collagen meshwork are closely related to the physiological alteration of the AC.

Bennighoff [32] first proposed that the collagen fibres in AC anchored to the subcondral bone and ran radically in the radical zone. The fibres curved in the transitional zone and continued to the superficial zone where they oriented predominantly in a direction parallel to the surface of articular cartilage for maximizing the tensile strength of articular surface. Since use of TEM [33], Benninghoff's collagen model in the transitional zone has been extensively debate but the collagen orientation in the subchondral bone and radial region are well accepted by most scholars [4,8,33]. Although most researchers agreed that the collagen fibres in the superficial have predominant parallel orientation to the articular surface, there were others reporting that the collagen structure in the superficial zone were much more complex [34] and the predominant parallel orientation to the articular surface were sometimes not prominent or absent [35].

Apparently, the collagen structure in the superficial zone found by this study is different to Benninghoff's observation and more complex than that of most 2D microscopic observations. However, the finding of the interwoven collagen network near the surface of normal AC in this study agrees with the study made more recently by atomic force microscopy (AFM) [36]. This collagen network is likely the source of the tensile property of the articular surface for wearing and shearing resistance. Particularly, the structure of the interwoven collagen bundles is ideal for resistance of the tensile and wearing stresses derived from unpredictable directions. The highly deposited proteoglycans in the normal AC, in contrast to the lower proteoglycan deposition in aged cartilage and the cartilage with surface disruption, may be also related to the structure of the interwoven collagen network, which can more effectively entrap the proteoglycans in the AC than the unidirectional collagen fibres.

**Figure 6:**
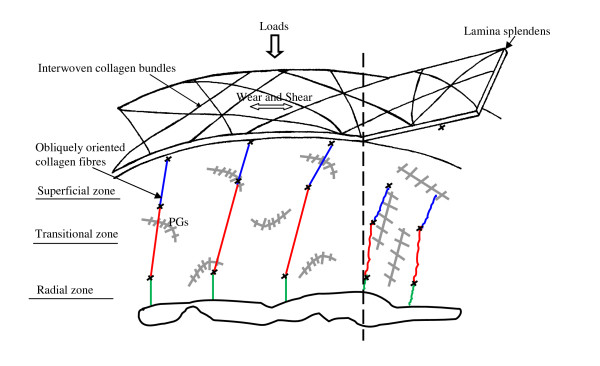
**A schematic structure of the collagen network in AC shows that the interwoven collagen bundles in the lamina splendens integrate the obliquely oriented collagen fibres and those in the deeper region to form a 3D collagen scaffold, which anchors to the subchondral bone.** It is well accepted that the 3D collagen scaffold arched on the subchondral bone of AC. It reinforces the swelling pressure of proteoglycan (PG) gel to provide the AC with loading capacities and considerable tensile strength to withstand for wear and shear stresses. Peeling off the lamina splendens where the interwoven collagen bundles reside reduces the wear and shearing resistance of the AC. It also leads to change of the osmotic pressure in AC and gradually release of PGs to the joint cavity. The tensile strength and lateral integrity of the interwoven collagen bundles permitted peeling off the most superficial layer from AC. This explains why torn articular surface occurs during excessive sports and exercises.

Peeling off the surface membrane corresponding to the lamina splendens is mainly attributed to the tensile strength of the interwoven collagen network and significant structural difference of this collagen network from the subjacent collagen fibres, as schematically shown in Fig 6. This basically agrees with the suggestion that the lamina splendens is a relatively independent layer with limited connections to the underlying cartilage [36]. It also explains why tore off articular surface occurs during sport accidents. Furthermore, the collagen fibres changed from oblique orientation to perpendicular orientation after peeling off the most superficial layer of AC could be associated to the remodeling of the osmotic pressure and subsequent expansion of the proteoglycans in the AC. Previously, oblique collagen fibres have be reported to run between the articular surface and subchondral bone [1], and they have further been suggested to be compatible to the requirement of entrapment of proteoglycans and strengthen the tensile properties [37]. Therefore, the oblique collagen fibres contained by normal AC may also have contributed to the normal mechanical function of AC. Conversely, the perpendicular collagen orientation found in majority of early OA cartilage may contribute little to retain proteoglycans and enhance the tensile property of the cartilage to wear and sharing stresses.

The correlation of the gradual disappearance of the interwoven collagen network to the progressive increase of the roughness of the AC surface shows the interwoven collagen network near the articular surface may play an important role in prevention of the initial lesion of AC surface and increase of the durability of the AC. This is consistent with the fact that loss of the most superficial layer of AC accelerates worn off AC [13]. Also, the similarity of the collagen structure in the cartilage physically peeled off the most superficial membrane and the cartilage with natural surface disruption indicates that the early pathological change in AC is closely related to the initial disruption of the collagen network near the articular surface. Elsewhere, the resembling of the collagen orientation between the aged cartilage and the cartilage with surface disruption explains the basic why the elders are more vulnerable to OA [20,38].

Although the interwoven collagen bundles exist near the articular surface, the collagen fibres in the superficial zone of the normal AC align predominantly in a direction spatially oblique to the articular surface (Fig 1b). This agrees with the traditional suggestions that the collagen fibres in the superficial have a predominant orientation to maximize the wear and shear resistance of AC [14]. The predominate collagen orientation may also attribute to measurement of the tensile strength of the superficial zone to be greater in one direction [15]. However, the relationship between the split line and predominate orientation of the collagen fibres in the superficial zone has not been confirmed in this study. Since the interwoven collagen bundles near the surface of normal AC, this study suggests if the split line represents the predominant orientation of the collagen fibres in the superficial, it would only represent the orientation of the oblique collagen fibres subjacent.

As shown in Figs 1(B) and 1(D), the 3D obliquely oriented collagen fibres can be translated as to align parallel to the articular surface while the interwoven collagen bundles can be easily over looked in a 2D *en face *image at large magnification. This suggests that the traditional view about the predominant collagen orientation in the superficial zone could be due to the limitation of the 2D microscopy for study of the 3D collagen fibres. Particularly, AC must be sectioned and dehydrated for many of electronic microscopic studies. The processes can cause significant changes to the collagen orientation in AC. After tissue dehydration, the interwoven collagen bundles are integrated with the subjacent oblique collagen fibres. Therefore, they have not been observed by electron microscopy.

The use of bovine cartilage as controlled healthy cartilage in this study is due to the unavailability of normal human cartilage. Since joints from different mammalian species have been suggested to be very similar in function and structure [39], this will not affect significantly to use the 3D imaging technique as a tool for examining the microscopic degeneration of the collagen network and early OA.

## Conclusion

This study examined the early physiological changes of AC in relation to the 3D collagen network. Therefore, a 3D histology, by which AC is not compromised of physical dehydrated and sectioned, has been developed to supplement the traditional histology for study of the 3D collagen network by means of monitoring lesions of articular cartilage and early OA.

Moreover, the fibre optic laser scanning confocal microscopy used in this study has an identical fibre imaging technique to confocal arthroscopy that has allowed studying the cellular structure of AC *in vivo *[28-30]. Although the staining technique used in this study is not clinical applicable, our current study on the investigation of clinical viable staining techniques for imaging the collagen and other micro-components of AC *in vivo *shows the potential of developing the 3D imaging technique to be a tool for assessing early OA and evaluating chondrocyte therapy technologies *in vivo*.

## Competing interests

The authors declare that they have no competing interests.

## Authors' contributions

JPW contributed the idea of use of the developed 3D imaging technique for study of physical status of articular cartilage, design and conducting the experiments, analyzing data and writing the manuscript. TBK participated in initiating the idea cartilage and proof read the manuscript. MHZ participated in design the experimental method and acknowledge of visual evaluation of articular cartilage's pathology. All authors read and approved the final manuscript.
